# A genome-wide association study of fetal response to type 2 porcine reproductive and respiratory syndrome virus challenge

**DOI:** 10.1038/srep20305

**Published:** 2016-02-05

**Authors:** Tianfu Yang, James Wilkinson, Zhiquan Wang, Andrea Ladinig, John Harding, Graham Plastow

**Affiliations:** 1 Department of Agricultural, Food, and Nutritional Science, University of Alberta, Edmonton, AB T6G 2P5, Canada; 2 University Clinic for Swine, Department for Farm Animals and Veterinary Public Health, University of Veterinary Medicine Vienna, Vienna 1210, Austria; 3 Department of Large Animal Clinical Sciences, Western College of Veterinary Medicine, University of Saskatchewan, Saskatoon, SK S7N 5B4, Canada

## Abstract

Control of porcine reproductive and respiratory syndrome (PRRS) is economically important for the swine industry worldwide. As current PRRS vaccines do not completely protect against heterologous challenge, alternative means of control, including enhanced genetic resilience, are needed. For reproductive PRRS, the genetic basis of fetal response to PRRS virus (PRRSV) infection is poorly understood. Genome-wide association studies (GWAS) were done here using data from 928 fetuses from pregnant gilts experimentally challenged with type 2 PRRSV. Fetuses were assessed for viral load in thymus (VLT), viral load in endometrium (VLE), fetal death (FD) and fetal viability (FV), and genotyped at a medium density. Collectively, 21 candidate genomic regions were found associated with these traits, seven of which overlap with previously reported QTLs for pig health and reproduction. A comparison with ongoing and related transcriptomic analyses of fetal response to PRRSV infection found differentially expressed genes within 18 candidate regions. Some of these genes have immune system functions, and have been reported to contribute to host response to PRRSV infection. The results provide new evidence about the genetic basis of fetal response to PRRSV challenge, and may ultimately lead to alternative control strategies to reduce the impact of reproductive PRRS.

Porcine reproductive and respiratory syndrome (PRRS) is one of the most serious threats to pig production worldwide^1^. Caused by PRRS virus (PRRSV), Family Arteriviridae^2^, it results in reproductive failure in sows, such as late-term abortion, premature delivery, stillborn or mummified fetuses, elevated preweaning mortality, and respiratory disease in neonatal and young pigs. Because of its widespread distribution, high mortality in infected herds, and poor performance in recovered herds^3^, this disease is economically very important for the swine industry. The estimated annual economic loss caused by PRRS in the US is more than 500 million US dollars, and its impact increased during the years 2005–2013^4^,^5^.

At present, developing effective vaccines against a wide range of PRRSV strains is still a challenge, as there is a gap in the knowledge of PRRS biology, pathogenesis and immunity^6^. Genomic tools may provide an alternative opportunity to explore the mechanisms behind PRRS, and to select animals resistant, or with reduced susceptibility to PRRSV^7^. For example, a major quantitative trait locus (QTL) on SSC4 associated with host response to PRRSV has been reported^8^ in a genome-wide association study (GWAS). Further analysis showed that estimated breeding values (EBV) based on this QTL were sufficiently accurate for potential use in animal selection to reduce the effects of PRRSV in growing pigs^9^. In addition, a QTL on SSC7 associated with reproductive traits and PRRS IgG antibody response was discovered, and was subsequently confirmed in an unrelated population^10^,^11^. However, since these important advances in our understanding of PRRS host responses were obtained from piglets post-weaning and dams, respectively, knowledge of the genetic basis of fetal response to PRRSV infection is still scarce.

To explore the mechanisms of reproductive PRRS, an experimental pregnant gilt challenge model (PGM) was undertaken^12^, and several phenotypic responses in dams and fetuses associated with PRRS severity were revealed^13^,^14^,^15^,^16^. The objective of the current study was to explore the genetic basis of fetal response to PRRSV infection, including viral load in thymus (VLT), viral load in endometrium (VLE), fetal death (FD) and fetal viability (FV), through GWAS using fetal samples from this challenge model.

## Results and Discussion

### Population Structure

**Figure 1:**
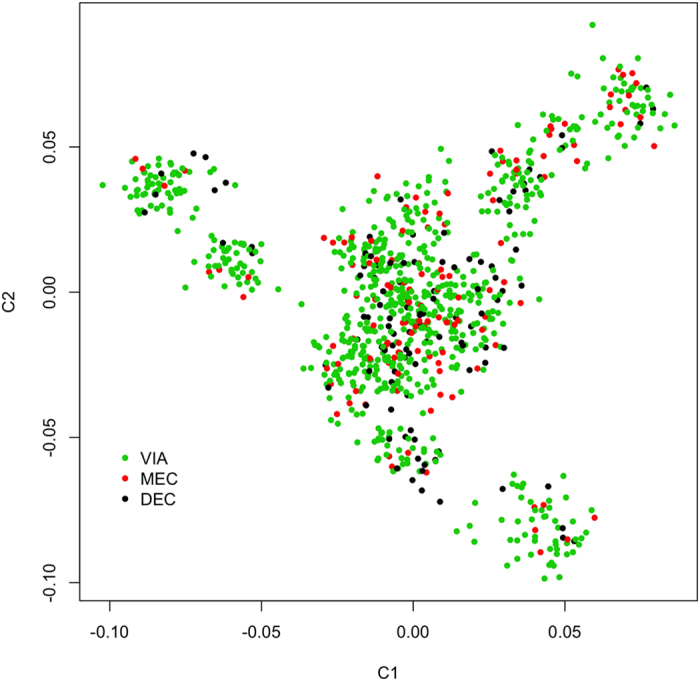
Population structure of the fetuses. The genetic distance was calculated using the genotype of 45,255 SNPs with Hamming distance. The plot was built with 2-D multi-dimension scaling (MDS), and presents the top 2 dimensions (C1 and C2) of the population structure. Colors of the points represent fetal preservation status: green for viable (VIA), red for meconium-stained (MEC), and black for decomposed (DEC). No substantial stratification was evident.

Figure 1 presents the result of multi-dimension scaling (MDS) and provides a visualization of the pairwise genetic distances among the fetuses. There was no evidence of population stratification based on the 2-dimensional plot. This conclusion was also supported by the principal component analysis (PCA) result. The eigenvalues for the top three principal components were only 2.49%, 2.45% and 2.26% of the summation of all eigenvalues, which implies a lack of population stratification.

### Association Results

In the regression analysis, 24 candidate SNPs were found associated with at least one of the four traits (VLT, VLE, FD, FV). Twenty-two could be mapped to the porcine genome and are located across 10 chromosomes. We also evaluated the contribution of these SNPs to the phenotypic variation, both individually and collectively. The results are grouped below by the four traits analyzed.

#### Viral Load Thymus (VLT)

**Figure 2:**
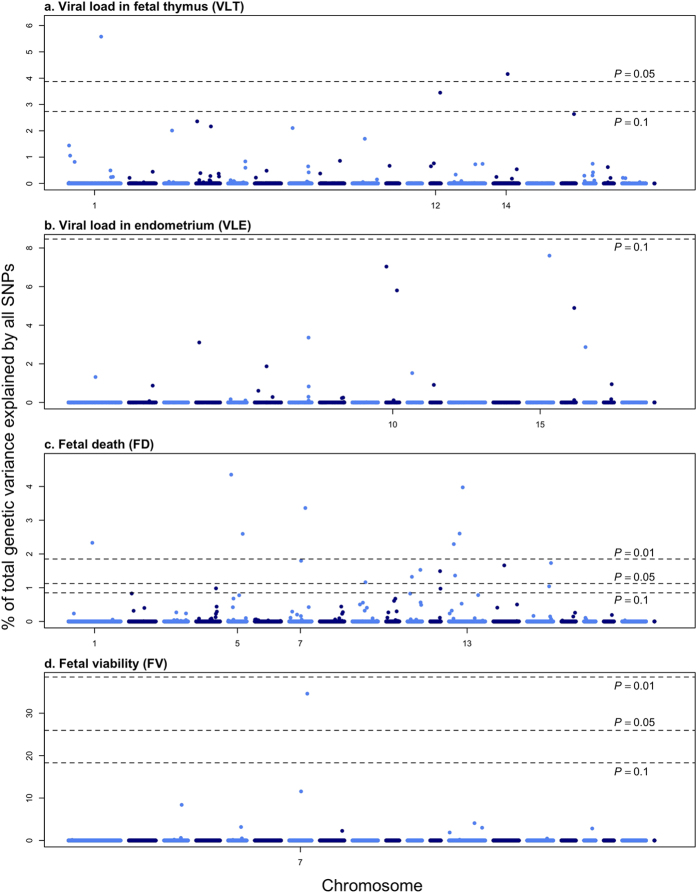
Manhattan Plot for viral load in fetal thymus (VLT), viral load in endometrium (VLE), fetal death (FD) and fetal viability (FV). The association analysis was conducted with the least absolute shrinkage and selection operator (LASSO). A generalized linear model was used. The Y-axes shows the percentage of total genetic variance (calculated as the variance of GEBV calculated using 45,255 SNPs) that can be explained by each single SNP. Thresholds corresponding to different empirical P-values were calculated with a 1,000-run permutation analysis.

Results of the associations between SNPs and VLT were summarized in Table 1 and Fig. 2a. One single nucleotide polymorphism (SNP) on each of *Sus scrofa* chromosome (SSC) 1 (ASGA0005344, *P* = 0.021) and SSC14 (DIAS0000654, *P* = 0.044) were significantly associated, and another SNP on SSC12 (ASGA0055300, *P* = 0.069) showed suggestive association with viral load. The three associated SNPs explained 5.58%, 4.16%, and 3.45% of σ^2^_g,total_ (total genetic variance explained by all SNPs passed quality control, see Methods section), respectively; and 13.0% collectively.

**Figure 3:**
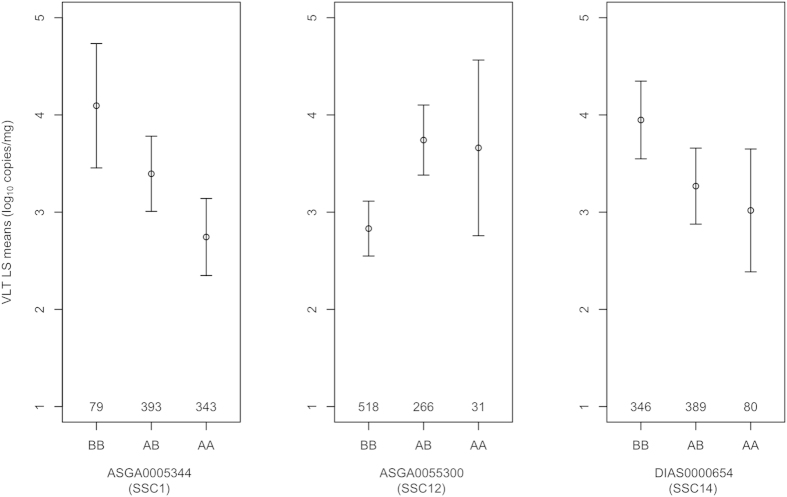
Least-square (LS) means of viral load in fetal thymus (VLT) for individuals with different genotypes for each of three SNPs showing significant (*P* < 0.05) or suggestive association (P < 0.1) with VLT. The error bars represent the 95% confidence interval. The numbers above the X-axis represent the total number of animals with that genotype.

For these three associated SNPs, a simplified linear model was used to investigate their genetic contribution. In this model, only two non-zero environmental effects, number of adjacent PRRSV-infected fetuses (nINF) and number of adjacent dead fetuses (nDEAD), and the three SNPs were fitted. All three SNPs were significantly associated with VLT (*P* < 0.001) in the regression. The least-square (LS) means for the SNPs were also calculated (Fig. 3). For the SNP ASGA0005344 on SSC1, the difference in LS mean between the two homozygous genotypes was 1.35 (log_10_ copies/mg). For the SNP ASGA0055300 on SCC12, the LS mean for the heterozygotes was obviously deviated from the average of the LS means for homozygotes, which implies the existence of a dominance effect. However, it should be noted that the number of fetuses with genotype AA is relatively small and the estimate of viral load may be an underestimate so that the true effect is additive.

**Figure 4:**
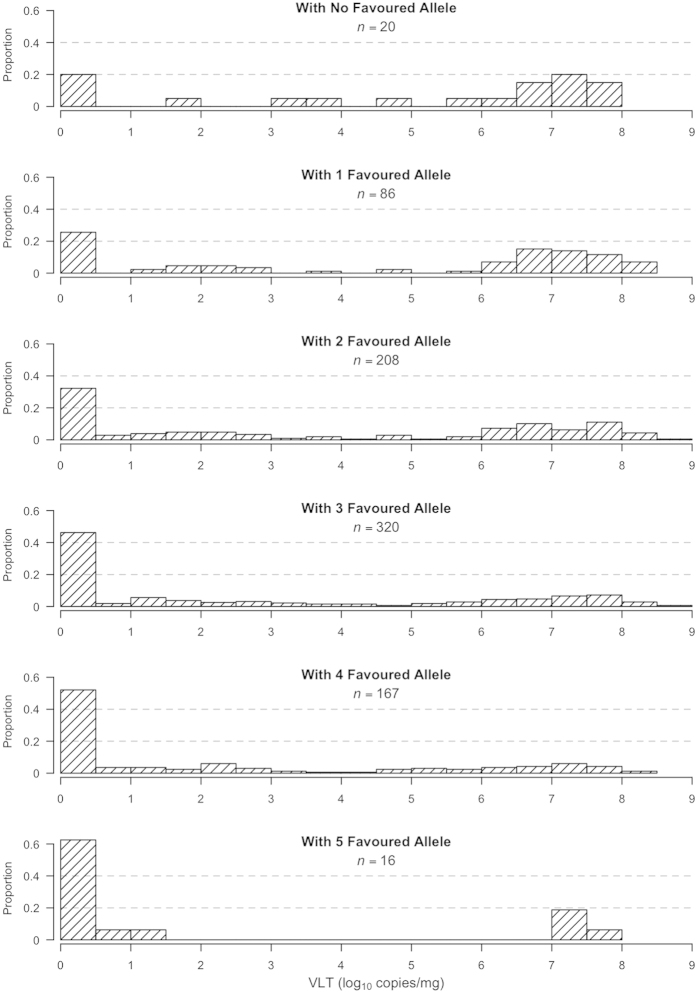
Distribution of viral load in fetal thymus (VLT) grouped by number of favoured alleles present in live fetuses. For each of the three SNPs showing significant (*P* < 0.05) or suggestive (*P* < 0.1) association with VLT, a favoured allele was determined. For each individual fetus, the total number of favoured alleles across the three SNPs was determined. The Y-axis represents the proportion of the fetuses (number of fetuses in that VL window divided by the number of all the fetuses in the group); X-axis represents PRRSV RNA concentration (logarithm 10 target copies per mg). The distribution shows the trend that a lower proportion of individuals had high VLT when more favoured alleles were present.

We also explored the distribution of the phenotypic values, to determine the interaction among the three SNPs. For each SNP, we determined the favoured allele, which was associated with a lower VLT. Fetuses were grouped based on how many favoured alleles they had across the three SNPs. The distribution for each group is shown in Fig. 4. It is evident that the proportion of fetuses with high VLT decreases when more favoured alleles were present in individual fetuses, which supports the conclusion that the effect of the three SNPs was additive.

#### Viral Load Endometrium (VLE)

No SNPs were significantly or suggestively associated with VLE (Fig. 2b; *P* > 0.1). However, the top SNP on SSC15 (ALGA0115095, located at ~134Mbp) was very close to a candidate SNP associated with FD.

#### Fetal Death (FD)

SNPs associated with FD are summarized in Table 2 and Fig. 2c. Sixteen SNPs were significantly associated with FD: seven SNPs with *P* < 0.01 and nine other SNPs with *P* < 0.05. Four additional SNPs had a suggestive association (*P* < 0.1). The *P*-values for each SNP were listed in Table 2. Two SNPs were unmapped in the latest genome map (build 10.2). In total, the 18 mapped candidate SNPs accounted for between approximately 1% and 4.4% of the genetic variance (σ^2^_g,total_) each and 35.4% of the variance overall, while the 7 most significant SNPs explain 20% of the genetic variance.

**Figure 5:**
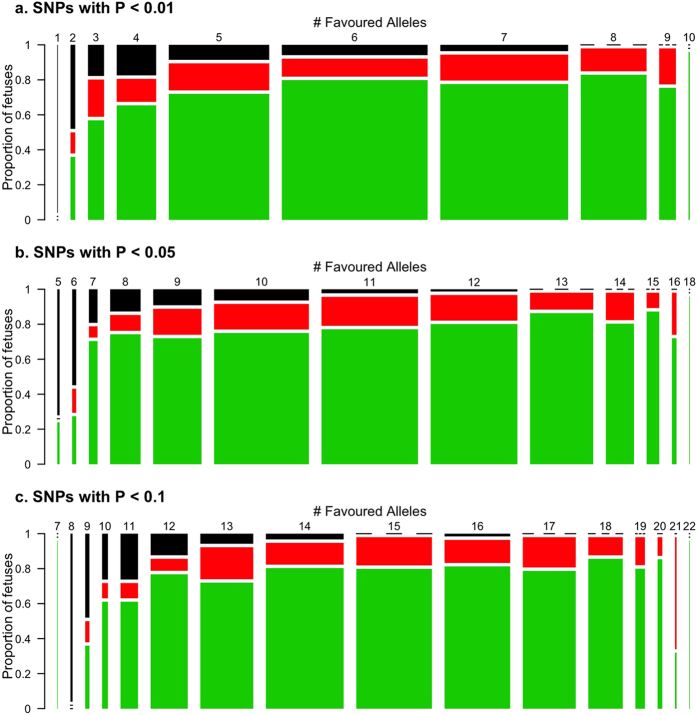
Distribution of fetal preservation status grouped by number of favoured alleles. For each of the SNPs significantly (*P* < 0.05) or suggestively (*P* < 0.1) associated with fetal death (FD), a favoured allele was determined. For each individual fetus, the total number of favoured alleles was determined, and the results displayed for set of SNPs based on their level of significance in the asociation analysis: a) 7 SNPs with *P* < 0.01, b) 15 SNPs with *P*-values < 0.05 or < 0.01, c) 18 SNPs with *P*-values < 0.1, < 0.05 or < 0.01. In each plot (A, B, C, *n* = 928), the area of each bar is proportional to the number of fetuses, with preservation status represeted by colour (green for viable [VIA], red for meconium-stained [MEC] and black for decomposed/dead [DEC]). The distribution shows the trend that the proportion of dead fetuses (DEC, black rectangles) decreases when more favoured alleles are present. However, the same trend was evident regardless of the number of SNPs included in the analyses (i.e. plots A, B, C look similar), and the number of favoured alleles did not affect the proportion of MEC fetuses (red rectangles), which remained relatively constant.

To explore if the associated genes interacted, the fetuses were grouped based on the number of favoured alleles they had and three plots were generated (Fig. 5) corresponding to sets of mapped SNPs: 1) seven SNPs with *P*-value lower than 0.01, 2) fifteen SNPs with *P*-value lower than 0.05, and 3) eighteen SNPs with *P*-value lower than 0.1. The proportion of viable (VIA), meconium-stained (MEC) and decomposed (DEC) fetuses was then compared for fetuses grouped by the number of favoured alleles they possessed. Whereas VIA fetuses appeared developmentally normal, MEC fetuses showed early signs of PRRS-related pathology, and DEC had died an estimated 3-5 days prior to termination^12^. Across all three plots, the proportion of DEC decreased as the number of favoured alleles increased. This trend was consistent regardless of the number of SNPs used in the analyses or their level of significance. This could be partly due to the low number of fetuses with an extreme number of favoured alleles in the dataset. Thus, a larger population may help to more accurately reveal the distribution for fetuses with extreme genotypes. In addition, the number of favoured alleles did not appear to affect the proportion of MEC fetuses, which support our assumption that the two traits, FD and FV, may each have a different genetic basis to a degree.

#### Fetal Viability (FV)

Results of GWAS for FV were summarized in Table 3 and Fig. 2d. Only one SNP on SSC7 was found to be significant (*P* = 0.018), which accounted for a considerable amount (34.0%) of total genetic variance (σ^2^_g,total_).

### Overlap with previously discovered QTLs

Some of the SNPs were found to be linked to candidate regions identified in previous studies. For example, the associated SNP located at 97-98 Mbp on SSC7 was very close to one of the candidate regions found associated with percentage of piglets born dead (PBD) in a commercial farm experiencing a PRRS outbreak^10^. Even though PBD was a trait measured at the litter-level and was associated with the genotype of sows, it is still noteworthy as both FD and PBD relate to fetal death. Based on a pigQTLdb search^17^, seven of the SNPs identified here were located within QTLs reported to be associated with pig disease resistance. One SNP associated with VLT is within a QTL of C3c serum concentration, which is a measurement of complement activity in innate immunity^18^. Six SNPs associated with FD are located in previously described QTLs that are related to leukocyte subset percentage, interferon level, Toll-like receptor level, pathogen count and reproductive traits^18^,^19^,^20^,^21^,^22^,^23^. More information about the pigQTLdb comparison is summarized in Table 4. However, we did not find any associated SNPs within the major QTL detected on SSC4 associated with viral load and growth rate in a PRRS nursery pig model^8^ nor the genomic region on SSC7 associated with antibody response in a PRRS reproductive outbreak^10^.

### Potential link with PRRS

An association suggests that there are mutations in a gene or genes in these regions that explain the observed phenotype. In order to investigate this further, we identified genes within the candidate regions to determine if there was support for a functional basis of the observation.

We defined 21 regions for the 22 candidate SNPs with map locations (SNP ASGA0055300 and M1GA0017106 are close together and therefore share the same candidate region). Then we searched for genes that were differentially expressed in an ongoing related transcriptomic analyses of fetal response to PRRSV infection (see Methods section). For one of the candidate regions on SSC1 (~197 Mbp), all four genes identified in this region were excluded due to their very low level of expression in fetal thymus (in the related transcriptomic analysis). Candidate regions on SSC13 (~64 Mbp) and SSC14 (~86 Mbp) do not harbour any differentially expressed genes in the transcriptomic analysis. However, we found differentially expressed genes in all of the other 18 candidate regions (see Supplementary Table S1 online). In the following sections, we propose some hypotheses about the potential functional links between those differentially expressed genes and the fetal response to PRRSV challenge.

#### Mechanisms of reproductive PRRS

Recent pathogenesis and immunological studies provide new information about the biology of fetal response to PRRSV infection. PRRSV has the capability to replicate in the fetus at any stage of gestation, as demonstrated by direct intra-fetal inoculation, but in a natural infection must first cross from dam to fetus via the placenta. The precise conditions required for transplacental infection are not fully understood, but are largely restricted to late in gestation^24^. In the current study, gilts were inoculated in late gestation (day 85 ± 1), which resulted in the death of about 40% of fetuses^12^. Previous studies suggested that PRRSV may cross the placental barrier through maternal macrophage migration^24^. Moreover, larger fetuses, which tend to have larger placentae, exhibit higher PRRS viral load and therefore appear to be more susceptible to PRRSV infection^14^. It seems likely that damage to the placental attachment site contributes to the reproductive pathology observed in PRRS. PRRSV-induced macrophage apoptosis in the placenta may lead to focal detachment and degeneration of the fetal placenta, resulting in fetal death^25^. However, events in the fetus itself may also influence the outcome of fetal infection. It has also been shown that both the status of adjacent fetuses, and the presence of PRRSV RNA (particularly at high levels in the fetus), are associated with fetal death, which suggests key roles for inter-fetal transmission and viral replication within fetuses^16^. Fetuses are immunocompetent as early as 79 days of gestation^26^. However, it is reported that immunity may remain dysregulated even in fetuses surviving *in utero* infection of PRRSV^27^,^28^. These results provide the basis for investigating the potential function of the regions identified here.

#### RIG-I pathway

Deconjugation of ubiquitin and ISG15 (IFN-stimulated gene product of 15 kDa) is involved in one of the modulation mechanisms by which PRRSV evades host immune responses^29^. As ISG15 plays an important role in antivirus defence through protein ISGylation, deconjugation of ISG15 leads to further inhibition of downstream signalling and innate immune responses, such as NF-kappa-B activation^30^. In one of our candidate genomic regions, we found gene *UBA7* (also known as *UBE1L*, encoding Ubiquitin-Like Modifier Activating Enzyme 7, located at ~36 Mbp on SSC13). The encoded enzyme catalyzes the conjugation of ISG15, and is critical in the protein ISGylation process^31^. These findings suggest the possibility that a mutation in this region of the genome may alter the expression of *UBA7* or the function of the encoded enzyme, and therefore modulate the host response to PRRSV.

#### Monocyte/macrophage lineage cells

PRRSV shows a strong tropism for monocyte/macrophage lineage cells, and it is reported that the differentiation and activation of these cells critically affect their susceptibility^32^. In our candidate regions, we found a set of genes critically involved in processes related to the differentiation and activation of monocytes to macrophages. They are gene *ACKR2* (also known as *CCBP2*, encoding atypical chemokine receptor 2, located at ~29 Mbp on SSC13), *CSF1* (also known as *M-CSF*, encoding macrophage colony-stimulating factor 1, located at ~121Mbp on SSC4), *MST1* (also known as *MSP*, encoding macrophage stimulating 1, located at ~35 Mbp on SSC13), and *MST1R* (also known as *RON*, encoding macrophage stimulating 1 receptor, located at ~36 Mbp on SSC13). Gene variants in these regions may result in changes to related pathways and ultimately to differences in the host’s innate and adaptive immune responses.

For example, ACKR2 is one of the receptors of CCL2^33^, chemokine (C-C Motif) ligand 2 (also known as MCP1). CCL2 is a key pro-inflammatory chemokine involved in the activation of monocytes^34^ and ACKR2 may act antagonistically due to its ability to scavenge extracellular chemokines including CCL2^33^. A previous study also suggested associations between circulating monocyte count and two missense variants within the gene *ACKR2* in humans^35^. In addition, Ladinig *et al.* previously reported for this challenge study that serum CCL2 levels in the gilts following PRRSV challenge were positively related to VL in serum and lung, but were not associated with the odds of fetal death^15^. A hypothesis based on these results is that a mutation within gene *ACKR2* results in fetal death, through the interference of ACKR2 in the functioning of CCL2.

It is reported that CSF1 is involved in the maturation and differentiation of monocytes into macrophages^36^, and CSF1 injections significantly increase macrophages and circulating monocytes in mice^37^.

Macrophage stimulating 1 (MST1) and its receptor (MST1R) establish the MSP-RON signalling system^38^ and mediate second messenger pathways within macrophages, including the Phosphatidylinositol-3-kinase (PI3K) pathway and mitogen-activated protein kinase (MAPK) pathway^39^, both of which are reported to be involved in host response to PRRSV^40^,^41^. The interaction between MST1 and MST1R may strongly modulate the production of interleukins (ILs), especially IL-12, IL-15 and IL-18^42^. All three ILs have been reported to modulate the activities of NK cells, such as their apoptosis, development and survival^43^,^44^.

#### Natural killer (NK) cells

It is reported that NK cell-mediated cytotoxicity was significantly decreased in pigs infected with PRRSV VR2332, a prototype type 2 PRRSV strain, and it appears to result in the suppression of IFN-γ production^45^. In our candidate regions, we found two genes that may be related to the activity of NK cells. They are *CXCR2* (encoding chemokine [C-X-C Motif] Receptor 2, located at ~133 Mbp on SSC15) and *NKTR* (encoding natural killer triggering receptor, located at ~29 Mbp on SSC13, although this gene was not differentially expressed in the transcriptomic analysis). These may play protective roles in PRRSV-induced immunosuppression, or be related to the modulation mechanism.

The product of *CXCR2* is a receptor that binds to a set of chemokines including IL-8 (also named CXCL8), which was reported to be a key factor in virus-induced selective chemotaxis of NK cells in humans^46^. A previous study suggested that IL-8 is one of the important cytokines involved in the clearance of virus from serum in PRRSV-infected pigs^47^. In the related cytokine profiling study by Ladinig *et al.* however, IL-8 level was not associated with viral load or fetal death, although it was significantly increased in PRRSV-stimulated peripheral blood mononuclear cells (PBMC) from infected pigs^15^.

NKTR is a cyclophilin-related protein, and the gene is exclusively expressed in NK cells^48^. The protein is believed to have an important role in NK cell cytotoxicity, and induce the production of IFN-γ^49^. As NK cell cytotoxicity and production of IFN-γ are both involved in PRRSV modulation of host innate immune response, it is tempting to speculate that NKTR may be part of this process.

#### T-cells

PRRSV is able to modulate host T-cell responses. One mechanism is reported to be upregulation of the frequency of Foxp3 + T-regulatory cells (T_reg_s), which secrete IL-10 and transforming growth factor β (TGFB) that suppress the host immune response^45^. A protein in the integrin family, αEβ7-integrin (also known as CD103), can help to retain T_reg_s and therefore may be key to this process^50^. We found the gene *ITGB7* (encoding β7-integrin, one of the two components of αEβ7-integrin) located in the candidate region at ~19Mbp on SSC5. Further *ITGB7* is one of the target genes of Foxp3 + , and it is possible that Foxp3 + has a direct effect on the expression of *ITGB7*^50^. In this case, *ITGB7* may be involved in the PRRSV-induced upregulation of Foxp3 + T_reg_s. On the other hand, β7-integrin is also related to the production of α4β7-integrin, which is involved in T-cell migration^51^. Gene *TMPO* (encoding thymopentin, located at ~90 Mbp on SSC5), may also have an impact on the PRRSV-induced modulation of host adaptive immune response, as thymopentin may be involved in the regulation of T helper cells, both Th1 and Th2, and their related cytokines, such as IFN-γ^52^.

#### IRF3/7 signalling

In a candidate region on SSC15 (~145Mbp), we also found genes encoding three members of the nuclear antigen SP100 family (SP100, SP110 and SP140), which may be related to the suppression of interferon (IFN) production by PRRSV. It is reported that PRRSV is able to inhibit the activation of interferon regulatory factor 3 (IRF3), and thereby suppress the synthesis of interferon-β (IFN-β)^30^. A previous study showed that SP100 may play a significant role in enhancing the production of IFN-β in IRF3/7 signalling^53^. Further evidence also suggests a role of the SP100 family in antiviral response through promyelocytic leukemia protein nuclear body (PML-NB)^54^, especially for viruses whose proteins localize to the nucleolus, such as PRRSV^55^.

#### Apoptosis and JNK signalling

Apoptosis is a critical process in the pathogenesis of PRRSV and the fetal response to PRRSV challenge. Evidence implies that PRRSV may be able to regulate the progress of apoptosis enhancing viral replication^56^. It was also proposed that the PRRSV-induced apoptosis at fetal implantation sites is a primary mechanism of fetal death^25^. Previous studies showed that c-Jun NH(2)-terminal kinase (JNK) pathway is critical to apoptosis, and that activation of the JNK pathway is required for PRRSV-induced apoptosis^57^. The JNK pathway can be activated by either of two MAPK kinases (MAP2Ks), and these two MAP2Ks can be activated by a total of fourteen MAPK kinase kinases (MAP3Ks)^58^. In our candidate genomic regions, we found two genes encoding kinases in this list. They are gene *MAP2K4* (encoding MAPK kinases 4, located at ~60 Mbp on SSC12) and *MAP3K12* (encoding MAPK kinase kinases 12, located at ~19 Mbp on SSC5). Neither showed differential expression in the transcriptomic analysis. MAP3K12 is not a kinase for MAP2K4, but activates another MAP2K, MAP2K7. We also found gene *MAPKAPK3* (encoding MAPK-activated protein kinase 3, located at ~36 Mbp on SSC13) in a candidate region, which was also not differentially expressed. MAPKAPK3 catalyzes the phosphorylation of heat shock proteins B1 (HSPB1)^59^, which is able to reduce the activity of JNK and thereby protect stressed cells from apoptosis^60^. Mutations of any of these three genes may have an effect on the activation of JNK and PRRSV-induced apoptosis, potentially leading to differential fetal responses, especially fetal death.

#### Response to secondary infection

In the candidate regions we also found a set of genes that function in the host response to pathogens, but have not been reported to be involved specifically in the response to single strand RNA viruses like PRRSV. These genes are *TLR9* (encoding toll-like receptor 9, located at ~38 Mbp on SSC13), *TREX1* (encoding the prime repair exonuclease 1, located at ~35 Mbp on SSC13), and *SLC11A1* (also known as *NRAMP1*, encoding solute carrier family 11 member 1, located at ~133 Mbp on SSC15).

As a member of the TLR family, TLR9 recognizes CpG motifs common to both bacterial and viral DNA and RNA:DNA hybrids^61^. The stimulation of TLR9 triggers the production of type I interferons (IFN), cytokines that play an important role in controlling viral infections^62^. Protein TREX1 is targeted as a microbial evasion strategy. This exonuclease can digest cytoplasmic single-stranded DNA decreasing its concentration thereby avoiding the stimulation of innate immune response^63^. Protein SLC11A1 protects macrophages and is reported to be involved in resistance to bacterial infection in pigs^64^.

Given that PRRSV is able to modulate the host immune response, increasing the host’s susceptibility to secondary infections^65^, these genes may have an indirect impact on fetal responses to PRRSV challenge, although they would not necessarily be expected to be associated with fetal outcome in this challenge model.

### Limitations and future work

Although the potential genomic regions and their genes and pathways discussed above may provide important clues for the fine mapping of specific causative mutations, additional research is required to confirm the roles of any these genes. An ongoing analysis of sequencing data is focusing on these genes to investigate potential candidate causative mutations. It should also be noted that although this GWAS was based on a relatively large number of fetuses, only one type 2 PRRSV strain and one termination time-point were used in the challenge experiment. Thus, the results may be specific to the experiment. Careful validation with different time-points and PRRSV strains, including type 1 PRRSV, are needed before generalizing the results. Some of this validation work is underway, including a new experiment with type 2 PRRSV using earlier time-point(s) for termination.

## Conclusions

In this GWAS of fetal response to PRRSV challenge, specifically fetal viral load (VLT, VLE) and fetal death and viability (FD, FV), we found 21 candidate regions located on 10 chromosomes, with four of these regions being on SSC13 and three regions on SSC7. Eighteen of the 21 candidate regions harbour genes showing differential expression associated with fetal PRRSV infection in a related transcriptomic study (Wilkinson *et al.* unpublished data), and 7 candidate regions overlap with previously reported QTLs involved in fetal health and host responses to pathogens. Within these regions, we found genes that are involved in a variety of immune processes, including cytokine signalling, leukocyte activities, and innate immunity, and a number of them are functionally linked to known PRRSV-related immune response pathways. The results may provide new evidence to help explain the genetic basis of the fetal response to PRRSV infection and may ultimately lead to alternative control strategies to reduce the impact of reproductive PRRS. However, it should be noted that only one type 2 PRRSV strain was studied; effects would need to be tested with other strains. Importantly, the major QTL identified in nursery pigs was shown to impact viremia and growth after infection with several type 2 strains^66^.

## Methods

### Animal resources

Samples and data used in the current study were obtained from a PRRS pregnant gilt challenge model, previously described in detail^12^. The experiment was approved by the University of Saskatchewan’s Animal Research Ethics Board and adhered to the Canadian Council on Animal Care guidelines for humane animal use (permit #20110102). In brief, 114 purebred Landrace gilts (Fast Genetics Inc., Spiritwood, Canada) were inoculated with type 2 NVSL 97-7895 PRRSV on gestation day 85 ± 1 and 19 similarly mock inoculated. All were humanely euthanized 21 days post inoculation (dpi). In total, 1422 fetuses obtained from the PRRSV-inoculated gilts were categorized based on their preservation status as: viable (VIA, *n* = 697), meconium-stained (MEC, *n* = 125), decomposed (DEC, *n* = 111), autolysed (AUT, *n* = 459), or mummified (MUM, *n* = 30). All AUT and MUM fetuses were excluded from the present analyses as poor DNA yield and quality prevented genotyping. VIA were alive until termination and externally normal, whereas MEC were alive but clearly showed pathologic changes. It is estimated that DEC died 3-5 days prior to termination based on their fetal size and primarily normal external appearance (more than 50% white skin, lack of generalized subcutaneous edema and emphysema).

### Phenotypic data

The viral load (VL; target RNA concentration per mg tissue) in fetal thymus (VLT) and endometrium (VLE) were measured in VIA, MEC and DEC fetuses using an in-house quantitative real-time PCR (qRT-PCR)^12^ targeted at a highly conserved region of the C-terminal end of ORF7 of NVSL 97–7895. Endometrium was collected from the umbilical stump of each fetus and included the adherent fetal placental layers. RNA was extracted from 10-20 mg tissue using the RNeasy extraction kit (Qiagen, Toronto, Canada) as per the manufacturer’s instructions. However, previous analysis showed that both VLT and VLE were considerably lower in DEC fetuses than in VIA and MEC, likely due to viral RNA degradation during the period of decomposition^12^. As the degree of RNA degradation was hard to measure and model, the VLT and VLE in DEC fetuses (*n* = 111) were excluded from the association analysis. The random errors of raw phenotypic values for VL traits were assumed to follow a lognormal distribution^1^, and were log-transformed (base 10) before the analysis (a phenotypic value of 0 was given to negative records). Two binary traits were also defined based on the fetal preservation: 1) fetal death (FD), where all DEC fetuses were coded as “dead” and all VIA and MEC fetuses were coded as “live”, and 2) fetal viability (FV), where all VIA fetuses were coded as “viable” and all MEC and DEC fetuses were coded as “non-viable”. We presumed that the genetic basis behind these two binary traits were different, so they were analyzed separately.

**Figure 6:**
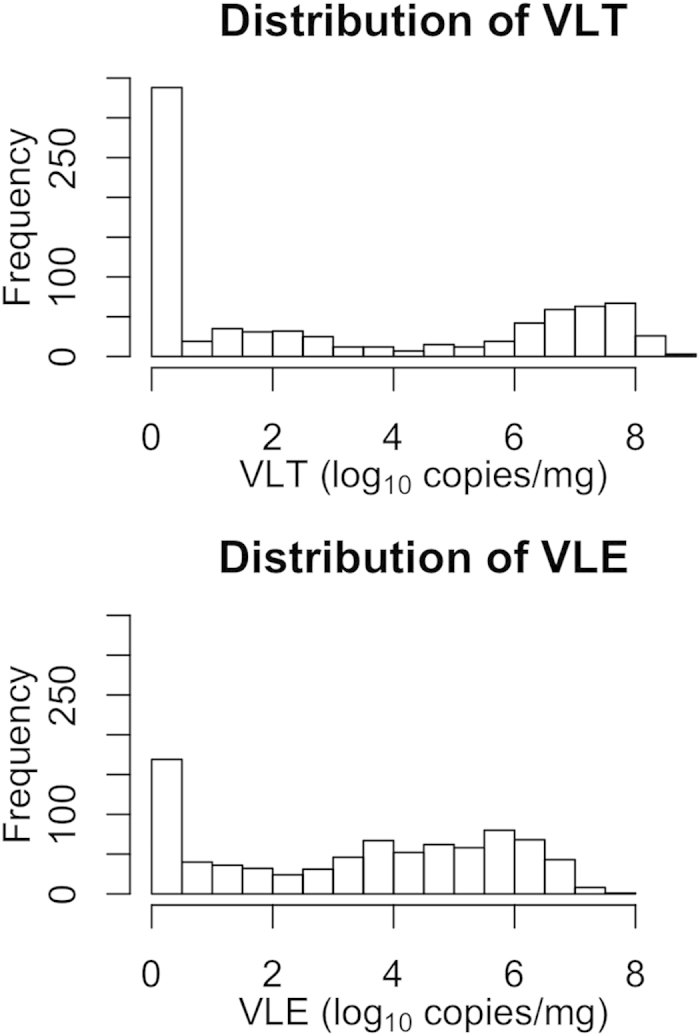
Distribution of viral load in fetal thymus (VLT) and endometrium (VLE) of live fetuses. Concentration of NVSL 97-7985 PRRSV RNA (log 10) per mg tissue measured by in-house quantitative real-time PCR.

The phenotype of VLT and VLE for genotyped fetuses was summarized in Table 5 and Fig. 6. It is noticeable that the VLT and VLE are not symmetrically distributed, but appear to follow a mixture of two distributions, one with high density near zero and the other roughly normal but spread over higher values.

Some other traits were also measured as potential environmental effects on fetal response to PRRSV. Inoculated dams were genotyped for the WUR10000125 SNP (Dam-WUR-SNP), which was associated with the SSC4 QTL^8^. Their serum viral load was also measured at day 0, 2, 6, 21 post-inoculation (Dam-VL-0, 2, 6, 21), using qRT-PCR and the same primers used for fetal viral load measurement. The area under the curve (Dam-VL-AUC0-21) was calculated from 0 to 21 dpi.

### Genomic data

In total, 928 fetuses with high DNA quality were genotyped using the PorcineSNP60 Genotyping BeadChip v2 (Illumina, San Diego, CA, USA) containing 61,565 SNPs. SNPs were filtered out when they: 1) had a call rate less than 90%; 2) had a minor allele frequency (MAF) less than 0.05; or 3) demonstrated a significant deviation from Hardy-Weinberg equilibrium (HWE) with a Chi^2^-value higher than 600. After the quality control, 45,255 SNPs remained in the dataset, with a missing call rate of 0.32%.

### Population Structure

To assess the potential impact of population stratification, the population structure of the fetuses was tested before the association analysis using Plink 1.90^67^ and R/rrBLUP^68^. Genetic difference among fetuses was measured as pairwise identity-by-state (IBS) Hamming distances in Plink, and a MDS analysis was conducted to construct the 2-D plot, which shows the first 2 dimensions of the population structure. A PCA by the eigenvalue decomposition of marker-base relationship matrix was also performed using R/rrBLUP. As there was no evidence that the population was substantially stratified, the population structure was not modelled in the following analysis.

### Model

A generalized linear model was used in the analyses:


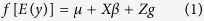


Two link functions (*f*) were used to transform the expectation of phenotypes (*E(y)*) to the linear predictor. For continuous phenotypes (VLE, VLT), an identity link function was used which was equivalent to a general linear model. For the two preservation traits (FD, FV), a logit link function was used to model the binary phenotype. *μ* was the intercept. *X* was the design matrix for environmental fixed effects, and *β* was the vector of environmental fixed effects, which included two litter factors (litter size and litter fetal mortality rate), three factors measuring maternal disease status (Dam-WUR-SNP, Dam-VL-21, Dam-VL-AUC0-21), three factors related to maternal uterine environment (relative fetal position within uterine horn increasing incrementally from tip to body [POS], nINF, nDEAD), and two other factors (fetal sex, experimental repetition/group). *Z* was the design matrix associated with SNP effects (*g*). In the design matrix, genotypes were coded as 1/0/-1 for genotype AA/AB/BB, respectively. All SNP effects (*g*) were treated as fixed effects.

The model was fitted using the least absolute shrinkage and selection operator (LASSO), which was reported to be appropriate for fitting fixed effects models in GWAS and genomic prediction^69^,^70^,^71^,^72^. The analysis was performed using R/glmnet package^73^, and the tuning parameter (λ) was selected to minimize the mean square error (MSE) in a 10-fold cross validation (CV) for each run. As the package does not accept design matrix with missing values, all missing genotypes were imputed as the overall average value of the marker. After the fitting of the model, the total genetic variance explained by all 45,255 SNPs (σ^2^_g,total_) and the genetic variance explained by each single marker (σ^2^_g,marker_) were calculated using the estimated SNP effects^74^.

### Permutation Test

Since R/glmnet has a very high computational efficiency^73^, the significance of those non-zero SNP effects were tested through permutation, by randomly shuffling the phenotype while keeping the genotype intact thereby destroying the association between the phenotype and genotype. Then, the permutated data were analysed with the same procedure to generate a null-distribution that was used to determine empirical test criteria underlying the null hypothesis^75^. One thousand runs were conducted for each trait. In each run, the σ^2^_g,marker_ for each marker was calculated. The highest σ^2^_g,marker_ in each run (“highest-σ^2^”) was used to construct the null distribution. For each SNP, its *P*-value was calculated as the proportion of highest-σ^2^ that were greater than the genetic variance explained by that SNP. When the proportion was equal to zero, it implied a *P*-value less than 0.001. The 99^th^ and 95^th^ percentiles (corresponding to an empirical *P*-value of 0.01 and 0.05) of the null distribution were used as the two critical values to test the result for that trait. Given the limited sample size in the current dataset (less than 1000 fetuses) and our relatively robust test strategy, the 90^th^ percentile (corresponding to an empirical *P*-value of 0.1) was also calculated to explore more potential SNPs.

### Transcriptomic analyses

To provide a functional context, GWAS results were compared with that of two differential gene expression experiments, in order to detect genes close to the candidate SNPs whose expression was altered in response to PRRSV infection. The results of the transcriptomic analyses came from a related study (Wilkinson *et al.* unpublished data) that investigated the fetal transcriptomic response to PRRSV infection, and used the same population of fetuses. In brief, for each of the two experiments, the gene expression profiles of two groups of fetuses (*n* = 12 per group) were compared. The first experiment compared fetuses from mock-inoculated control gilts (CON) to viable, qRT-PCR positive fetuses from PRRSV-inoculated gilts (INF). The second experiment compared INF fetuses to viable, uninfected (qRT-PCR negative) fetuses from PRRSV-inoculated gilts (UNINF). All differentially expressed genes located within a 4Mbp window (2Mbp upstream or downstream) of any of the candidate SNPs were identified. More details about these genes can be found in Supplementary Table S1 online. The results may provide support for possible QTLs in those regions, and aid in the identification of the causative SNP if it affects gene expression. Although those genes without differential expression may also contain causative polymorphisms, this functional analysis was mainly focused on those differentially expressed genes, as they are more likely to play a role in the fetal response to PRRSV.

The location of all SNPs were based on *Illumina Pig 60k SNPs mapped to pig genome assembly 10.2* 76,77. The searching of genes near candidate SNPs was performed using BioMart, an online service that integrated the information from top bioinformatic databases, and R/Bioconductor package biomaRt, an R interface for BioMart^78^,^79^.

## Additional Information

**How to cite this article**: Yang, T. *et al.* A genome-wide association study of fetal response to type 2 porcine reproductive and respiratory syndrome virus challenge. *Sci. Rep.* **6**, 20305; doi: 10.1038/srep20305 (2016).

## Supplementary Material

Supplementary Table S1

## Figures and Tables

**Table 1:** Top SNPs associated with viral load in fetal thymus (VLT).

Marker ID	Location		*P*	σ^2^_g,marker_/σ^2^_g,total_ (%)
	Chromosome	Position (bp)		
ASGA0005344	1	197,479,988	0.021	5.58
ASGA0055300	12	59,921,056	0.069	3.45
DIAS0000654	14	85,991,839	0.044	4.16

The locations of SNPs were based on Illumina Pig 60k SNPs mapped to pig genome assembly 10.2^76^. Empirical *P*-values were calculated with a 1,000-run permutation analysis. σ^2^_g,marker_ means the genetic variance explained by the single marker, and σ^2^_g,total_ means the total genetic variance explained by all 45,255 SNPs.

**Table 2:** Top SNPs associated with fetal death (FD).

Marker ID	Location		*P*	σ^2^_g,marker_/σ^2^_g,total_ (%)
	Chromosome	Position (bp)		
MARC0003250	1	144,155,620	0.005	2.33
ASGA0021980	4	119,682,308	0.073	0.98
MARC0042986	5	18,071,917	<0.001	4.35
ASGA0026553	5	87,726,880	0.002	2.60
DRGA0007745	7	71,531,707	0.012	1.80
ASGA0035226	7	97,430,037	< 0.001	3.36
ALGA0053793	9	78,113,024	0.048	1.16
ALGA0061607	11	26,560,250	0.036	1.32
MARC0089129	11	77,708,385	0.026	1.53
M1GA0017106	12	59,174,296	0.027	1.49
MARC0077450	12	61,568,245	0.074	0.97
ALGA0069106	13	28,741,495	0.005	2.29
ASGA0057175	13	36,517,366	0.033	1.36
ALGA0070448	13	63,937,862	0.002	2.60
ALGA0070951	13	82,891,929	<0.001	3.97
M1GA0018773	14	65,422,608	0.018	1.66
MARC0055746	15	132,949,028	0.065	1.04
ALGA0087932	15	144,250,933	0.015	1.73
MARC0076503	–	–	0.047	1.19
ALGA0032154	–	–	0.067	1.02

The locations of SNPs were based on Illumina Pig 60k SNPs mapped to pig genome assembly 10.2^76^. Empirical *P*-values were calculated with a 1,000-run permutation analysis. σ^2^_g,marker_ means the genetic variance explained by the single marker, and σ^2^_g,total_ means the total genetic variance explained by all 45,255 SNPs.

**Table 3:** Top SNPs associated with fetal viability (FV).

Marker ID	Location		*P*	σ^2^_g,marker_/ σ^2^_g,total_ (%)
	Chromosome	Position (bp)		
DRGA0008048	7	109,279,352	0.018	34.6

The locations of SNPs were based on Illumina Pig 60k SNPs mapped to pig genome assembly 10.2^76^. Empirical *P*-values were calculated with a 1,000-run permutation analysis. σ^2^_g,marker_ means the genetic variance explained by the single marker, and σ^2^_g,total_ means the total genetic variance explained by all 45,255 SNPs.

**Table 4:** QTLs overlapping with candidate regions.

	QTL Information		
SNP ID(Associated Trait)	Chromosome	Span (bp)	Trait
ASGA0021980 (FD)	4	119,299,797–120,509,810	CD4-positive; CD8-negative leukocyte percentage; CD4-positive leukocyte percentage
ASGA0026553 (FD)	5	85,756,451–90,130,141	Interferon-γ level
MARC0089129 (FD)	11	73,438,559–78,480,320	CD4-positive leukocyte percentage
ASGA0057175 (FD)	13	36,281,164–36,541,751	Mummified pigs
DIAS0000654 (VLT)	14	83,873,248–93,372,131	C3c concentration
M1GA0018773 (FD)	14	53,465,935–81,745,465	Salmonella count in liver and spleen; Salmonella count in liver
MARC0055746 (FD)	15	135,157,314–149,797,711	Toll-like receptor 2 level

The QTL information was based on pigQTLdb^17^. Only QTLs spanning no more than 30Mbp were listed in the table, as those QTLs spanning a larger region provide limited support. Associated traits include viral load measured in fetal thymus (VLT) and fetal death (FD).

**Table 5:** Summary of fetal viral load measured in fetal thymus (VLT) and fetal viral load measured in endometrium (VLE) for genotyped fetuses.

Trait	No. of observations	Mean	Standard Deviation	Minimum value	Maximum value
VLT	817	3.06	3.20	0	8.80
VLE	817	3.35	2.35	0	7.61

The PRRS virus concentration (target RNA copies/mg) has been log-transformed (base 10). Zeros were given to negative records.
